# ESPRIT-Forest: Parallel clustering of massive amplicon sequence data in subquadratic time

**DOI:** 10.1371/journal.pcbi.1005518

**Published:** 2017-04-24

**Authors:** Yunpeng Cai, Wei Zheng, Jin Yao, Yujie Yang, Volker Mai, Qi Mao, Yijun Sun

**Affiliations:** 1 Shenzhen Institutes of Advanced Technology, Chinese Academy of Sciences, Shenzhen, China; 2 Department of Computer Science and Engineering, The State University of New York at Buffalo, Buffalo, New York, United States of America; 3 Department of Microbiology and Immunology, The State University of New York at Buffalo, Buffalo, New York, United States of America; 4 Department of Epidemiology, University of Florida, Gainesville, Florida, United States of America; 5 Department of Biostatistics, The State University of New York at Buffalo, Buffalo, New York, United States of America; University of Canterbury, NEW ZEALAND

## Abstract

The rapid development of sequencing technology has led to an explosive accumulation of genomic sequence data. Clustering is often the first step to perform in sequence analysis, and hierarchical clustering is one of the most commonly used approaches for this purpose. However, it is currently computationally expensive to perform hierarchical clustering of extremely large sequence datasets due to its quadratic time and space complexities. In this paper we developed a new algorithm called ESPRIT-Forest for parallel hierarchical clustering of sequences. The algorithm achieves subquadratic time and space complexity and maintains a high clustering accuracy comparable to the standard method. The basic idea is to organize sequences into a pseudo-metric based partitioning tree for sub-linear time searching of nearest neighbors, and then use a new multiple-pair merging criterion to construct clusters in parallel using multiple threads. The new algorithm was tested on the human microbiome project (HMP) dataset, currently one of the largest published microbial 16S rRNA sequence dataset. Our experiment demonstrated that with the power of parallel computing it is now compu- tationally feasible to perform hierarchical clustering analysis of tens of millions of sequences. The software is available at http://www.acsu.buffalo.edu/∼yijunsun/lab/ESPRIT-Forest.html.

This is a PLoS Computational Biology software paper.

## Introduction

Genome sequencing is a common tool in biological and biomedical research. In the past few years, the data generation capacity of high-throughput sequencing technology has increased dramatically at a speed exceeding Moore’s law [1] and with sharply reduced cost. For example, the latest Illumina HiSeq 2500 platform can produce 600 million sequences of 300bp in length (2×150bp, 180GB data in total) in 40 hours. The rapid accumulation of genomic information represents a valuable source to significantly expand biological knowledge but meanwhile poses a serious challenge for data analysis, demanding new computational algorithms for efficient data processing.

Shotgun and amplicon sequencing are currently two major sequencing approaches. Amplicon sequencing, which sequences a specific genome region identified by primers, is a powerful tool for in-depth analysis of phylogenetic and evolutionary details, especially for ecology studies of viruses [2] and microbial communities including bacteria [3], fungi [4] and planktons [5], and the analysis of germline and somatic mutations of human (e.g., cancer cells [6] or immune cells [7]). For example, metagenomics of human gut microbiota, where amplicon sequencing of 16S rRNA gene serves as a major probing tool, has become an exploding research area listed as one of the top ten breakthroughs in 2013 by the Science magazine [8].

Usually, the first major step in processing amplicon sequencing data after quality control is to bin sequences into taxonomic or genotypic units, which forms the basis for performing ecological statistics and comparative studies [9]. Existing methods can be generally classified into taxonomy-dependent approaches, where sequences are anno- tated against a reference taxonomy database, and taxonomy-independent approaches [10], where sequences are clustered into operational taxonomy units (OTUs) based on pairwise similarities without using external references (thus also called *de novo* binning [9]). Since the main goal of amplicon sequencing is usually to explore uncharted biospheres where a significant portion of genetic material is contributed by previously unknown taxa, taxonomy-independent analysis is often the preferred, if not the only, choice.

A dozen of methods have been proposed for the *de novo* binning of amplicon sequences. Yet, the computational burden of generating clusters from massive sequence data remains a serious challenge, and only a few algorithms are able to handle millions of sequences. To accurately measure the similarity between sequences and sequence clusters, sequence alignment is usually employed, which is computationally very expensive and represents a bottleneck for clustering algorithms. Hierarchical clustering (HC) is one of the most widely used approaches for sequence binning [11], which usually exhibits quadratic time and space complexity (when pairwise alignment is employed) or even higher complexity (when multiple alignment is used) due to the need of generating a pairwise similarity matrix. Various data preprocessing heuristics (e.g., removing redundant reads, using kmer distances as a filter to remove sequence pairs with large genetic distances, splitting a distance matrix) were recently proposed thatproven to be very effective in reducing the computational time of a clustering process. However, these heuristics do not fundamentally change the nature that HC is a quadratic-time algorithm. As a tradeoff between computational efficiency and accuracy, several heuristic methods including Cd-hit [12] and UCLUST [13] were recently proposed that employ greedy clustering instead of hierarchical clustering to reduce the computational complexity associated with sequence comparison. Several benchmark studies have shown that the use of greedy clustering can lead to considerable loss in clustering quality compared with hierarchical clustering [14–16]. However, due to the high computational complexity of hierarchical clustering, heuristic methods remain to date the *only* computationally feasible solution to process massive sequence datasets such as those generated by the human microbiome project (HMP) [17].

In our previous work, we developed a sequence clustering algorithm called ESPRIT-Tree that performs hierarchical clustering efficiently with subquadratic complexity in both time and space while maintaining an accuracy comparable to the standard method [18]. It is capable of handling one million sequences with an average length of ∼250bp within less than one day on a single personal computer. Several large-scale benchmark studies have been performed by us and others on real-world datasets that identified ESPRIT-Tree as one of the best algorithms for taxonomy- independent analyses [14–16, 19, 20]. However, the single-thread nature of ESPRIT-Tree poses a limitation on its data processing capacity. As high performance computing clusters are becoming widely accessible, parallel computing is a feasible approach to further exploiting the computational power of subquadratic hierarchical clustering to handle even larger sequence datasets. However, efficient parallelization of hierarchical clustering is inherently difficult and only a few attempts [21–23] have been made using traditional hierarchical clustering involving quadratic complexity. Among them, HPC-Clust [24], ESPRIT [25], CRISPY [26] and LAHDC [27] are currently the only few parallel solutions for sequence clustering. HPC-Clust resorts to profile-based alignment to reduce computation burden, which we have shown to lead to inferior clustering quality when dealing with uncharted taxa [14]. Furthermore, it merely parallelizes the distance-calculation step, but not the cluster-merging step which takes a significant portion of the execution time on large datasets. Another major issue with HPC-Clust is that its memory usage grows quadratically with respect to the number of sequences, which makes it incapable of processing millions of sequences. ESPRIT and CRISPY are essentially quadratic-time algorithms, which are accurate but computa- tionally inefficient and take a couple of days to process one million reads. We recently proposed LAHDC as an attempt of parallelizing ESPRIT-Tree, which speeds up the algorithm by partitioning the data into subsets that can be clustered in parallel, with minor loss in clustering quality. However, more sophisticated solutions are still desired to efficiently parallelize the hierarchical clustering steps without sacrificing the cluster- ing quality. On the other hand, to the best of our knowledge there is still no prior work done on the parallelization of subquadratic hierarchical clustering methods.

In this paper we propose a new parallel algorithm, ESPRIT-Forest, which is able to handle the problem of hierarchical clustering of tens of millions of sequences accurately, with subquadratic time and space complexity and a good scalability with respect to the number of CPUs. We utilize the basic concept behind ESPRIT-Tree that organizes sequences into a pseudo-metric based partitioning (PBP) tree structure to achieve sub-linear time complexity when searching for nearest neighbors, and propose a new multiple-pair merging algorithm that performs search on a PBP tree for parallel construction of clusters. As such, multiple computing threads can be used to perform hierarchical clustering on the same PBP tree without interfering with each other. As a result, the clustering procedure can be accelerated efficiently by using multiple processors. With the newly developed algorithm, we successfully performed a hierarchical clustering analysis of the HMP data, currently one of the largest published microbial 16S rRNA amplicon sequencing dataset, within less than 40 hours on a small computing cluster. As currently there is no hierarchical clustering method available to process such large datasets, this work represents a significant progress in algorithm development to overcome the computational bottleneck of microbial OTU binning.

Hierarchical clustering is an essential tool for in-depth analyses of sequence data, including not only sequence binning but also sequence alignments [28, 29] and construction of phylogenetic trees [30, 31]. Thus, the algorithm proposed in this paper can help to boost the computational capacity of many sequence-processing pipelines, which in turn could enhance the contributions of bioinformatics to knowledge discovery in sequencing-based studies.

## Design and implementation

### Subquadratic time hierarchical clustering using PBP tree

Hierarchical clustering is one of the most frequently used clustering methods in sequence analysis. It works by iterating a process of picking up a pair of samples or clusters with the minimal distance among all possible pairs of samples or clusters and then merging them into a new cluster, until only one single cluster is left or the distances between clusters all exceed a given threshold. Let $D=\{x_{1},x_{2},\ldots ,x_{N}\}$ be a dataset containing *N* samples (sequences), $\left\{S_{i}\right\}$ be the obtained clusters and $D(S_{1},S_{2})$ be a binary function defining the distance between any two clusters, a hierarchical clustering algorithm is formally described in Algorithm 1.

**Algorithm 1**: Hierarchical Clustering

1 **Input**: $D={\left\{x_{n}\right\}}_{n=1}^{N}$, stop criterion *d*_*up*_, distance function *D*(⋅);

2 **Initialization**: $S_{n}=\left\{x_{n}\right\},1\leq n\leq N,\Omega_{0}=\left\{1,\ldots ,N\right\}$, *k* = 0;

3 **repeat**

4  $\left\{a,b\right\}={\text{argmin}}_{i,j\in \Omega_{k},i\neq j}D\left(S_{i},S_{j}\right)$;

5  *k* = *k*+1;

6  $S_{N+k}=S_{a}\cup S_{b}$;

7  Ω_*k*_ = Ω_*k*−1_ ∪ {*N*+*k*}\{*a*, *b*};

8 **until** |Ω_*k*_| = 1 or $\min_{i,j\in \Omega_{k},i\neq j}D\left(S_{i},S_{j}\right)\geq d_{up}$;

9 **Output**: A set of generated clusters $\left\{S_{1},\ldots ,S_{N+k}\right\}$ and a set of existing clusters Ω_*k*_.

**Fig 1:**
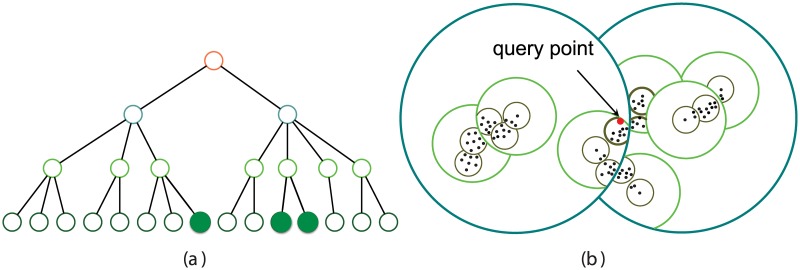
(a) A toy example of a PBP tree and (b) its corresponding partitioning of a dataset and a space. The colors indicate different levels of nodes and their corresponding hyper-spheres. The leaf nodes are omitted in the tree. When searching for the nearest neighbor of a point (large red dot), only a small number of sibling hyper-spheres (filled circles) need to be explored.

The most time-consuming step in hierarchical clustering is the identification of the closest cluster pairs (line 4 in Algorithm 1) which requires computation of all N*(*N* − 1)/2 distance pairs in the first iteration and *O*(*N*) updates in each subsequent iterations. In our previous work [18] we proposed a data structure called pseudo-metric based partitioning (PBP) tree to enable fast searching of the closest pairs by making only a small number of distance comparisons. A PBP tree is an ordered, equal-depth tree that partitions a sequence space with a series of hyper-spheres. Each node of a PBP tree represents a hyper-sphere in the space with a selected sample as the center. The nodes in the tree are then organized as a hierarchical structure with multiple levels, with all nodes at the same level having an equal sphere radius and nodes at the upper level having a larger radius than those at the lower levels. Each leaf node at the bottom level has a radius of zero and is created for each sample in a dataset, with the sample point as the center. Each node at the same level is assigned an order number, according to the order at which the node is created in the tree. For a given node, a parent node is chosen from the nodes at the upper level whose hyper-sphere region covers the center of the given node, and has the minimal order number among all candidates. Finally, a root node is defined as the parent of all top-level nodes, without a defined center or radius. Fig 1 illustrates a toy example of a PBP tree and how it partitions a dataset in a space.

By organizing sequences in a PBP tree and performing a branch-and-bound searching, we can find the nearest neighbor of any given sequence efficiently and determine the closest pair in sublinear time, avoiding the computation of the entire pairwise distance matrix. On the other hand, insertion (deletion) of a sequence into (from) the PBP tree takes almost constant computational time. Furthermore, after each modification, the new closest pair can be updated on-the-fly with sub-linear computation time. Hence, by iteratively merging the closest pair into one cluster and inserting the new cluster back to the PBP tree, we are able to perform hierarchical clustering very efficiently. In practice, ESPRIT-Tree achieved a computational complexity of $O(N^{1.2})$ to $O(N^{1.3})$ on various benchmark datasets (*N* is the number of sequences) with the best clustering accuracy among existing methods. A detailed description on the branch-and-bound searching and on-the-fly maintenance of a PBP tree can be found in [18].

Despite its efficiency, ESPRIT-Tree can only be executed on one computer processor due to the restriction of finding and merging closest pairs one at a time, which limits its application to ultra-large-scale data. In this paper we address this issue by allowing multiple cluster merging operations to be executed on one single PBP tree in parallel, which is described in detail below.

### Multi-point parallel clustering on PBP tree

**Fig 2:**
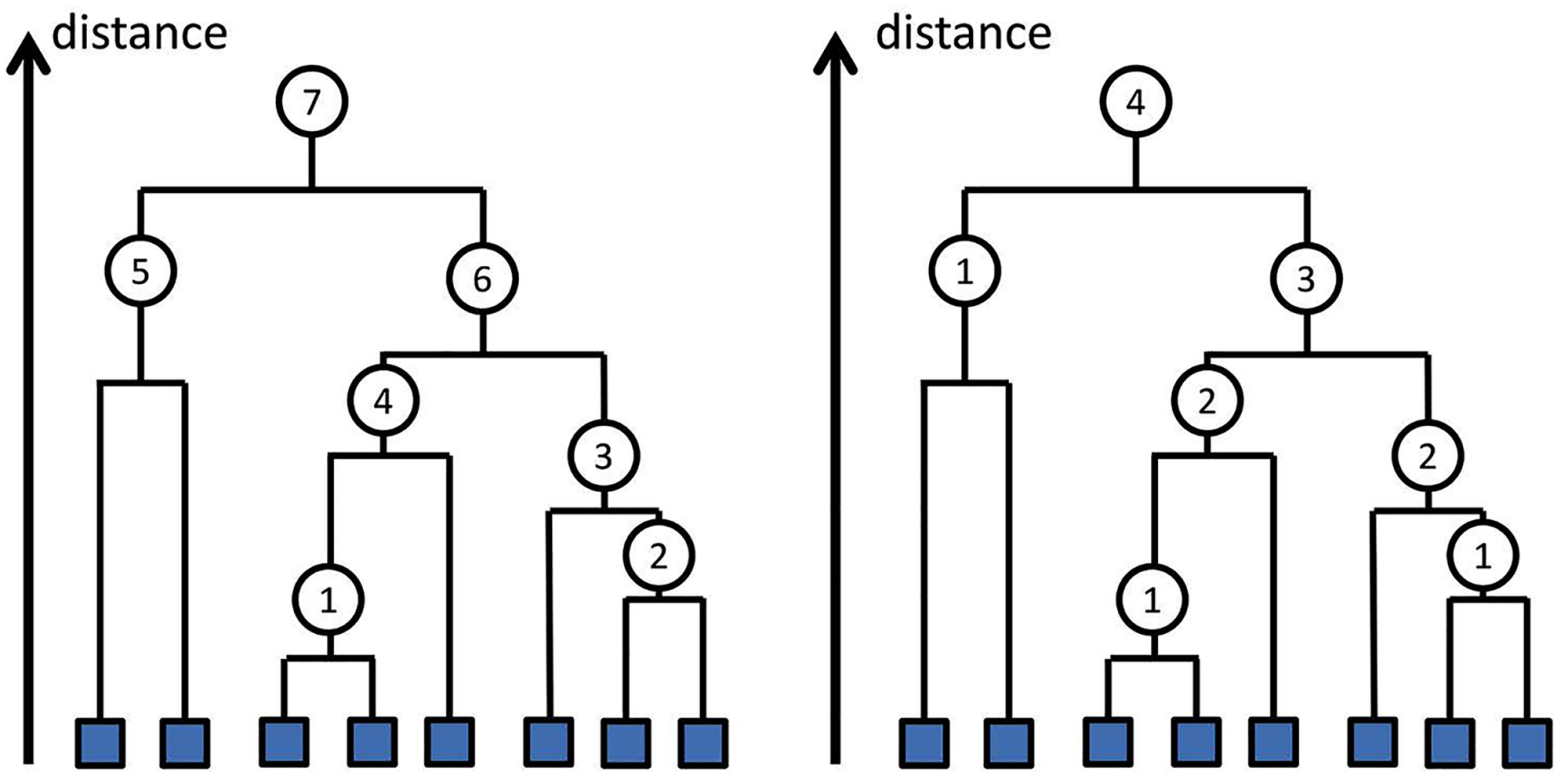
A toy example illustrating single-point (left) and multiple-point (right) hierarchical clustering by parallelizing uncorrelated operations. Each filled box represents a sequence and each circle represents a cluster-merging step. The numbers in the circle denote the order of merging operations. The merging orders may change when switching from single-point to multi-point clustering.

Parallelizing the hierarchical clustering process is difficult because for each merging operation it is required to keep track of *all* previous merging results, which restricts the capability of multiple threading. We tackle this problem by exploiting the implicit independency among clustering steps. A new cluster-merging criterion is proposed that strictly replicates the results of the standard hierarchical clustering method but at the same time decouples and re-orders the cluster merging steps, enabling hierarchical clustering to be executed on several merging points in parallel. Fig 2 illustrates the basic idea of parallel merging with a toy example showing that some clustering steps can be carried out simultaneously without violating the merging rules used in the standard hierarchical clustering process.

The key challenge of parallel merging is to design a clustering scheme that produces results identical to that generated by standard hierarchical clustering, which is important in order to preserve clustering quality. We demonstrate below that by using a proper merging criterion, multi-point clustering can achieve a clustering result equivalent to that of standard single-point hierarchical clustering. Specifically, in each step, we can merge all pairs {*a*, *b*} that satisfy *NN*(*a*) = *b* and *NN*(*b*) = *a* (Here *NN* denotes the nearest neighbour), without violating the rule used conventional hierarchical clustering. The modified algorithm is described in Algorithm 2.

We prove that, in the case of sequence clustering and many other normal situations, a clustering result generated by Algorithm 2 is always consist with one generated by Algorithm 1. The formal statement and the proof of the theorem are provided in S1 Methods. With Algorithm 2 we can extend our previous ESPRIT- Tree algorithm to a parallel version called ESPRIT-Forest. Table 1 depicts the overall framework of our new algorithm, ESPRIT-Forest, and the comparison with the previous single-thread version, with the parallel execution parts underlined. It should be noted that, although the key modification (Step 3) itself is not parallelized, it is the basis for achieving high parallelization efficiency. Without the modified criterion, the parallelization step (Step 5) will be inefficient due to too few deliverable tasks.

**Table 1:** Comparison of the overall frameworks of ESPRIT-Tree and ESPRIT-Forest. The parallel execution parts are underlined.

ESPRIT-Tree		ESPRIT-Forest	
1.	Construct a PBP tree and insert all samples	1.	Construct a PBP tree in each computing node and insert all samples
2.	Find the nearest neighbor (NN) for each sample	2.	Find the nearest neighbor (NN) for each sample in parallel
3.	Find the closest pair from a NN list, delete the pair from the PBP tree and merge them to form a new cluster	3.	Select *all pairs* {*a*, *b*} satisfying *NN*(*a*) = *b* and *NN*(*b*) = *a*, delete each pair from the PBP tree and merge each of them into a new cluster
4.	Insert the new cluster into the PBP tree	4.	Insert all new clusters into the PBP tree
5.	Update the NN list for the new cluster and all affected clusters through NN search	5.	Update the NN list for the new clusters and all affected clusters through NN search in parallel
6.	Repeat Steps 3–5 until the stopping criterion is met	6.	Repeat Steps 3–5 until the stopping criterion is met

**Algorithm 2**: Multi-point Hierarchical Clustering

1 **Input**: $D={\left\{x_{n}\right\}}_{n=1}^{N}$, stop criterion *d*_*up*_, distance function *D*(⋅);

2 **Initialization**: $S_{n}=\left\{x_{n}\right\},1\leq n\leq N,\Omega_{0}=\left\{1,\ldots ,N\right\}$, *k* = 0;

3 **repeat**

4  Set $\Phi =\{\left\{a_{i},b_{i}\right\}|\;{\text{NN}}_{\Omega_{k}}\left(S_{a_{i}}\right)=b_{i},{\text{NN}}_{\Omega_{k}}\left(S_{b_{i}}\right)=a_{i},D\left(S_{a_{i}},S_{b_{i}}\right)<d_{up}\}$;

5  **for** *i* ← 1 **to** |Φ| **do**

6   *k* = *k*+1;

7   $S_{N+k}=S_{a_{i}}\cup S_{b_{i}}$;

8   Ω_*k*_ = Ω_*k*−1_ ∪ {*N*+*k*}\{*a*_*i*_, *b*_*i*_};

9 **until** |Ω_*k*_| = 1 or $\min_{i,j\in \Omega_{k},i\neq j}D\left(S_{i},S_{j}\right)\geq d_{up}$;

10 **Output**: A set of generated clusters $\left\{S_{1},\ldots ,S_{N+k}\right\}$ and a set of existing clusters Ω_*k*_.

### Code implementation

#### Programming model

A master-slave programming model was adopted in the implementation of ESPRIT- Forest. The master thread takes charge of all samples and distributes tasks to the slave threads, while the slave threads are launched by the master thread to execute required operations and become inactive after that. In a shared-memory architecture (SMA) computer, this can be easily implemented by using parallel programming modules such as OpenMP [32]. However, shared-memory computers with many CPU cores are expensive and currently many high-performance computing (HPC) centers do not have a shared-memory computer with more than 32 CPU cores. Therefore, in order to ensure the scalability of our method, we implemented it using a symmetric multi-processor (SMP) model where multiple computing nodes do not access the memory of each other, but communicate via a message passing interface (MPI). The code is implemented in C++ using OpenMPI [32] and OpenMP at the same time.

An appealing property of ESPRIT-Forest is that the PBP tree does not need to be shared or synchronized among computing nodes, because the possible discrepancy of the constructed trees will not lead to inaccuracy in NN search, provided that the properties of the PBP trees are maintained. In practice, each node reads the entire sequence data, constructs a PBP tree independently, and modifies the tree by following the same instructions from the master node, which leads to almost identical tree structure (with small discrepancy caused by floating point errors). During the clustering procedure, in each iteration, the master node launches a list of NN queries and assigns each query task to a computing node (including the master node itself). Each computing node responds to the query by executing NN search tasks in its assignment list, and returns the results to the master node. The master node then determines which clusters should be merged and what new queries should be launched next. After cluster merging operations are made on the master node, it broadcasts the changes to the slave nodes and each computing node makes changes to its own PBP tree accordingly. In this way, the computing nodes are weakly coupled and the communication costs are minimized, which guarantees the efficiency of parallelization.

#### Tie-breaking

An issue not considered in the above framework Table 1 is that there may be ties in the searching of the nearest neighbors. For example, for three clusters *a*, *b* and *c*, it may hold that *NN*(*a*) = *b*, *NN*(*b*) = *c* and *NN*(*c*) = *a*, with equal NN distances. In this case, the above proposed framework may fail to discover mergeable pairs, which causes the algorithm to be inefficient or even fail. To avoid this problem, a tie-breaking operation is carried out in each iteration that scans all unpaired clusters and check if they can be paired. For an unpaired cluster *a* and its counterpart *b* = *NN*(*a*), if *b* is also unpaired and *D*(*b*, *NN*(*b*)) ≥ *D*(*a*, *b*), then *NN*(*b*) is forced to be *a* to make a mergeable pair.

#### Delayed updating of nearest neighbors

With the proposed multi-point merging criterion, a large number of merging and NN updating are generated in each iteration, which makes it possible for efficient paralle- lization. However, a potential problem is that some NN update requests may not be necessary. For example, if we search the NN of a cluster *a*, resulting in a solution *NN*(*a*) = *b*, but later *b* is merged with another cluster and we have to search the NN for *a* again, the former search effort is actually wasted and brings additional computation burden. To avert this issue, we design a delayed updating scheme that postpones a NN search until it has to be carried out. Due to the non-decreasing property of hierarchical clustering, when the NN of a cluster becomes invalid (i.e., merged into a new cluster), the old NN distance can be used as an estimate of the lower bound of a new NN distance. Two heaps [33] are created in the master nodes, with one storing a sorted list of NN pairs to be merged, and the other storing a sorted list of clusters for which a search needs to be launched to update NN information. The master node inspects each candidate NN pair in the first heap. If the distance of the candidate pair is smaller than the estimated NN distance of all pre-updated clusters, the pair can be merged regardless of the incomplete NN information. If there is at least one mergable pair in the heap, the master node carries out the merging first without launching update requests; otherwise, it launches a predefined number of updates (20 times the number of the used CPU cores) to ensure that the clustering steps can be continued with a balanced workload.

#### Pipeline and data pre-processing

ESPRIT-Forest inherits the same pipeline of ESPRIT and ESPRIT-Tree, which takes sequences in FASTA format as inputs and performs three major operation steps: pre-processing, hierarchical clustering and statistical analysis. The pre-processing step is identical with ESPRIT and ESPRIT-Tree, which merges redundant sequences and filters out sequences with a poor match with their primers or having lengths deviated too much from the average length. Although chimera checking is not implemented in ESPRIT-Forest, it can be performed efficiently with existing tools such as UCHIME [34]. We should point out that although ESPRIT-Tree/ESPRIT-Forest does not pay lots of efforts on data pre-procesing, it is a stand-alone algorithm which can be used in couple with other data preprocessing techniques or as a basic building block in a pipeline to further speed up a clustering process. The processed sequences are fed to hierarchical clustering module and two types of clustering outputs are generated. The first output is the complete hierarchical tree described in a tetrad table format with each line representing a clustering step. The second is the cluster partitioning of sequences at various given distance thresholds, which can be customized by the users. Finally, a statistical analysis module inherited from ESPRIT/ESPRIT-Tree performs ecological statistical analysis including the ACE estimates, the CHAO1 estimates and the Rarefaction curves [25] on user-defined distance levels.

## Results

We tested the proposed ESPRIT-Forest algorithm on several large-scale real-world datasets acquired from 16S rRNA sequencing of human microbiome, and compared with three leading sequence-clustering algorithms, ESPRIT-Tree, UPARSE (the latest version of UCLUST [35]) and HPC-Clust. For UPARSE and UCLUST, the default parameters are used per the authors’ suggestion. The tests were performed on a small HPC cluster with 32 nodes, each equipped with two Intel Xeon E5-2660 8-core CPUs and 128GB memory. Up to 256 (16×16) cores were used in the test.

### Scalability test

**Fig 3:**
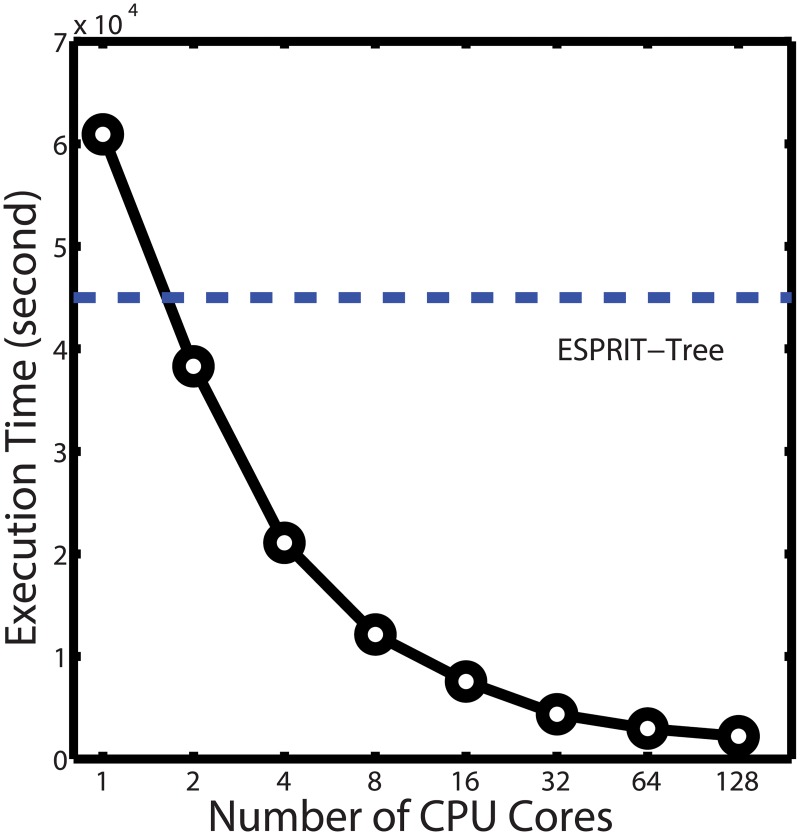
Execution time of ESPRIT-Forest performed on a human gut microbiome dataset using a varying number of CPU cores ranging from 1 to 128. The clustering termination criterion was set to 85% sequence similarity. For comparison, the execution time of ESPRIT-Tree is also reported.

We first tested the parallelization efficiency of ESPRIT-Forest. To this end, a human gut microbiome dataset from an obesity study [36] was used, which contains 1.1M reads (470K unique sequences) with an average length of 233bp. Fig 3 depicts the running time of ESPRIT-Forest performed on 1 to 128 CPU cores. For comparison, the running time of the single-thread ESPRIT-Tree algorithm is also reported. With 128 cores, ESPRIT-Forest finished the analysis of the entire dataset in 2,218 seconds, more than 20 times faster than ESPRIT-Tree. With only 2 CPU cores, ESPRIT-Forest surpasses ESPRIT-Tree in speed. This demonstrates a good scalability of the algorithm to utilize the power of parallel computing. In the single-core environment, ESPRIT-Forest is slower than ESPRIT-Tree. This is because in order to reduce the communication cost in parallel execution, we force a large number of NN updates (20 times the number of CPU cores) to be executed in a batch. Consequently, some unnecessary NN updates are carried out in the single-core case. This, however, is a necessary trade-off.

We also tested HPC-Clust on the same dataset. However, even with 128GB memory per node and at a very high similarity threshold 0.99, HPC-Clust failed to complete the distance calculation and crashed after about one hour of execution. This suggests that HPC-Clust is not suitable to handle large datasets. In order to make the comparison feasible, we sampled a subset of 300K reads (93,269 unique reads) from the dataset and executed each algorithm on it. Table 2 reports the execution time of ESPRIT-Forest, ESPRIT-Tree, HPC-Clust and UPARSE. ESPRIT-Forest and HPC-Clust were executed on 32 CPU cores. We can see that even after excluding the time used for sequence alignments, the clustering step of HPC-Clust still took nearly twice the total time of ESPRIT-Tree (it used only one CPU core) and is 15 times slower than ESPRIT-Forest. Although UPARSE is faster, it is a greedy method and the clustering result is quite coarse as demonstrated in the next subsection.

**Table 2:** Execution time of four clustering methods performed on a subset of the human gut microbiome data. ESPRIT-Forest and HPC-Clust (including the INFERNAL alignment method it used) were executed on 32 CPU cores.

Method	ES-Forest	ES-Tree	HPC-Clust (incl. align)	HPC-Clust (excl. align)	UPARSE
Time	251s	1942s	217h	3695s	70s

### Benchmark on clustering quality

In addition to computational efficiency, clustering quality is another important consideration when evaluating a clustering method. Although we have proved in theory that our parallelization method should produce results compliant with the single-thread version, the multi-solution nature of hierarchical clustering and the approximation steps taken in ESPRIT-Tree may lead to some random fluctuations. Here we carried out an experiment to demonstrate that parallelization does not affect the quality of clustering outcomes. It should be noted that the purpose of the experiments carried out in this section is to confirm that the performance of ESPRIT-Forest is similar to ESPRIT-Tree in statistical sense using other methods as anchors, rather than to compare ESPRIT-Forest with previous methods. Comprehensive comparison of 16S rRNA clustering or OTU picking methods is quite a sophisticated and controversial topic, which is out of the scope of this paper. A brief discussion on this issue will be provided later in the paper.

The benchmark test protocol proposed in [14] was used. Briefly, a benchmark database comprising sequences that can be reliably annotated to a known taxon was used as the ground truth. The annotation was done using a BLAST [37] search against the RDP-II [38] database (not the RDP classifier), whose annotation was enhanced by the TaxCollector [39] tool to achieve precise species-level labeling. A test dataset was created by random sampling without replacement from the benchmark database. The test dataset was fed into a clustering algorithm, and the clustering results generated at various similarity thresholds were then compared with the true annotation using the normalized mutual information (NMI) index. The peak NMI score was used to measure the clustering quality. To remove statistical variations, twenty test sets were randomly generated in each run and box-plots were created to compare the quality of different clustering results. In this paper, four benchmark databases were used, two from the human gut microbiome dataset [36], one from the ELDERMET dataset and one from the saliva subset of the HMP project.

**Fig 4:**
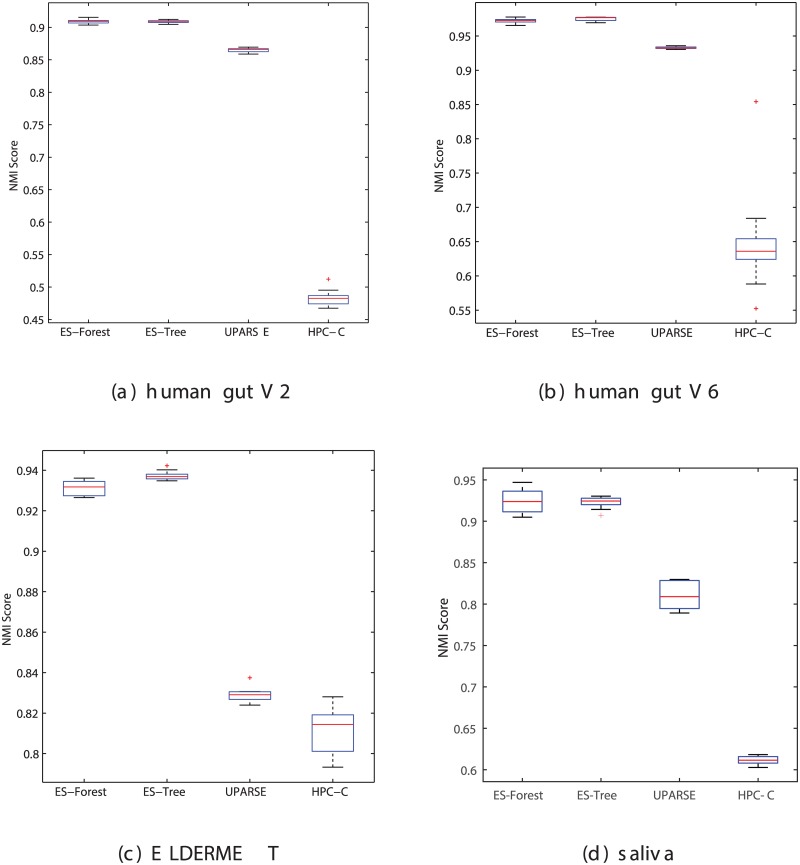
Comparison of clustering quality of ESPRIT-Forest, ESPRIT-Tree and UPARSE performed on benchmark datasets using the species annotation as ground truth. (a) NMI scores calculated on human gut V2 dataset. (b) NMI scores calculated on human gut V6 dataset. (c) NMI scores calculated on ELDERMET dataset. (d) NMI scores calculated on HMP Saliva dataset.

**Fig 5:**
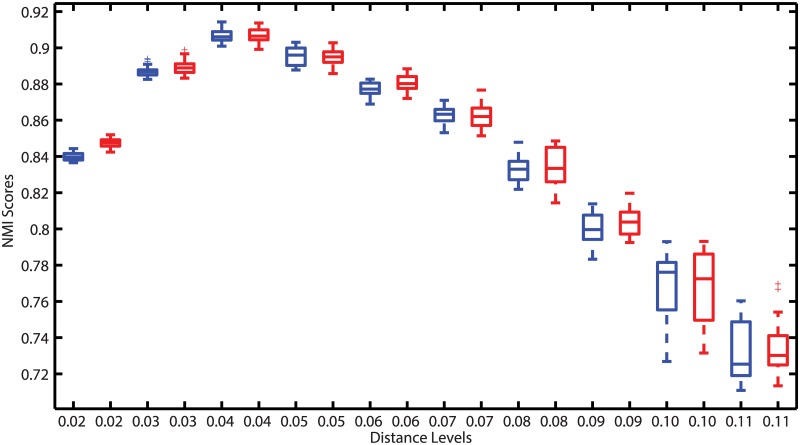
Comparison of clustering quality of ESPRIT-Tree (red) and ESPRIT-Forest (blue) on various distance cut-offs on the human gut V2 dataset. We see that the results of both algorithms agrees but with small variations caused by randomness in clustering.

Fig 4 reports the peak NMI scores of ESPRIT-Forest, ESPRIT-Tree, UPARSE and HPC-Clust performed on four benchmark datasets, respectively, averaged over twenty runs. ESPRIT-Forest achieved statistically identical quality compared with ESPRIT-Tree (p-value > 0.2 based on Student’s t-test) and both performed significantly better than UPARSE (p-value < 10^−22^). This result shows that hierarchical clustering indeed performs much better than heuristic methods, which is consistent with previous work [14–16, 19, 20]. It also empirically verifies the theoretical result that multi-point clustering does not change the nature of a clustering algorithm. To further verify this, the NMI scores achieved for ESPRIT-Tree and ESPRIT-Forest on the first dataset with different distance cut-offs are depicted in Fig 5. We see that the results of both algorithms agree on various distance levels. Moreover, despite the lengthy time consumed in HPC-Clust, it lead to inferior clustering quality due to the profile-based alignment method employed.

### Tests on large-scale real world datasets

To further demonstrate the power of ESPRIT-Forest on processing large amplicon sequencing datasets, we applied the algorithm to the HMP and ELDERMET datasets [40]. The HMP dataset comprises 19.8 million raw reads sequenced from different hypervariable regions of the microbial 16S rRNA gene, including 9.6 million reads from the V1-V3 regions, 9.6 million reads from the V3-V5 regions, and 0.7 million reads from the V6-V9 regions. Since clustering sequences from different regions makes no biological sense, we partitioned the data into three subsets based on their targeted regions and clustered the two largest subsets. The ELDERMET dataset comprises 9.0 million raw reads sequenced from the V2-V3 regions. Although several studies have analyzed the above datasets [40–42], only taxonomy-dependent approaches were used. To the best of our knowledge, no taxonomy-independent analysis has been performed before using a hierarchical clustering method.

Table 3 reports the detailed information of the datasets and the execution time required for ESPRIT-Forest (using 256 CPU cores) to complete the analysis. By using parallel computing, the proposed algorithm is able to finish the hierarchical clustering analyses of all datasets in less than 40 hours. This experiment suggests that with the power of parallel computing it is now computationally feasible to perform hierarchical clustering of tens of millions of sequences. Considering that heuristic greedy methods with low clustering quality was the only available clustering method capable of handling extremely large sequence data, this work thus represents a significant progress in algorithm development to overcome the computational bottleneck of microbial OTU binning.

**Table 3:** Execution time of ESPRIT-Forest performed on HMP and ELDERMET datasets. The clustering termination criterion was set to 85% sequence similarity. ESPRIT preproc was used to remove low-quality reads before clustering analysis.

Datasets	Data size	No. raw reads	No. high-quality reads	No. unique reads	Ave. length (bp)	Exec. time (ES-Forest)
HMP V1-V3	5.5GB	9,547,737	7,678,585	5,063,052	482	33h
HMP V3-V5	5.4GB	9,547,362	7,747,712	4,277,831	483	38h
ELDERMET	2.5GB	8,989,448	7,068,272	3,826,973	254	8h

## Avalability and future directions

The ESPRIT-Forest software is available at http://www.acsu.buffalo.edu/∼yijunsun/lab/ESPRIT-Forest.html, including the source code of both MPI and OpenMP implementations with pre-compiled executable codes. The software is an open-source program following the OSI certificated Adaptive Public License (https://opensource.org/licenses/APL-1.0). With the rapid development of sequencing technologies, the computational bottleneck hinders the efficient mining of acquired large data sets. In this paper, we described a novel efficient parallel hierarchical clustering algorithm, ESPRIT-Forest, to tackle the computational bottleneck associated with the taxonomy-independent analyses of amplicon sequencing data. Using a small HPC cluster, the proposed algorithm successfully processed the entire HMP dataset, one of the currently largest published 16S rRNA sequence dataset, within less than 40 hours, which suggests that ESPRIT-Forest is capable of handling most of the existing amplicon sequencing datasets. The source code of the algorithm is made available so that it can be incorporated with existing tools and pipelines.

One severe challenge for sequence clustering for very large scale problems is that the entire dataset has to be loaded into the memory of every computation node and processed together. Hence the capacity of a single node limits the size of the problem that can be solved. In one of our previous work we proposed LAHDC [27] which enables partitioning of sequence data into subsets and each computation node conquers a subset separately, but with slight loss in clustering quality. In order to handle very large datasets, the advantages of LAHDC and ESPRIT-Forest should be fused, which would be one of our future direction.

Another striking challenge brought by the advent of next-generation sequencing technology is the rapid growth of sequence length. Many third-generation sequencing technology claims to have a long read length of 10*k* to 100*k* base-pairs, which made sequence alignments more time consuming. To cope with this, parallelizing the Needleman-Wunsch algorithm for pairwise sequence alignments with GPU computation would be a feasible solution, which would require more sophisticated design on the programming architecture and the computer hardware infrastructure in order to support GPU-CPU hybrid computing.

The problem of identifying microbe taxonomic groups (OTUs) from 16S rRNA sequencing has been investigated for many years with tons of algorithms and tools proposed but debates still remain on various scopes [43, 44]. Due to the limitation of human knowledge on microbial communities, establishment of reliable ground truth becomes very difficult and evaluations of OTU picking often lead to controversial results with different datasets or criteria used. Moreover, recent studies have suggested that alignments and clustering methods are not the only major factors that affect the clustering quality, and sequence preprocess or denoising would largely affect the final results [45, 46], which further increases the complication of this issue. For this reason, in current stage it is hard to settle the complete OTU picking problem with a single pipeline. Alternatively, a stand-alone programs that can be flexibly incorporated with existing pipelines and provides incremental improvements would be favored. On the other hand, some recent studies have suggested that a dynamic distance cut-off accounting the phylogentic topology of the entire microbe community would improve the quality of OTU picking [47, 48]. Compared with greedy-based approaches, our hierarchical clustering approach provides more detailed information about the entire community, thus leaving more potential of improvements for advanced OTU-picking methods in the future. Moreover, since hierarchical clustering plays a fundamental role in many analytical procedures other than sequence binning, such as phylogenetic tree construction [49], sequence alignments [50], sequence database construction and searching [51], etc., our general approach can be used to boost the efficiency of other sequence analysis tools.

## Supporting information

S1 Methods Theoretical proof for the equivalence of parallel hierarchical clustering to sequential ones. (PDF)

## References

[1] Sboner A, Mu XJ, Greenbaum D, Auerbach RK, Gerstein MB. The real cost of sequencing: higher than you think! Genome Biology. 2011;12(8):125 10.1186/gb-2011-12-8-125 21867570 PMC3245608

[2] Beerenwinkel N, Zagordi O. Ultra-deep sequencing for the analysis of viral populations. Current Opinion in Virology. 2011;1(5):413–418. 10.1016/j.coviro.2011.07.008 22440844

[3] Sogin ML, Morrison HG, Huber JA, Welch DM, Huse SM, Neal PR, et al Microbial diversity in the deep sea and the underexplored “rare biosphere”. Proceedings of the National Academy of Sciences. 2006;103(32):12115–12120. 10.1073/pnas.0605127103 PMC1524930 16880384

[4] O’Brien HE, Parrent JL, Jackson JA, Moncalvo JM, Vilgalys R. Fungal community analysis by large-scale sequencing of environmental samples. Applied and Environmental Microbiology. 2005;71(9):5544–5550. 10.1128/AEM.71.9.5544-5550.2005 16151147 PMC1214672

[5] López-García P, Rodríguez-Valera F, Pedrós-Alió C, Moreira D. Unexpected diversity of small eukaryotes in deep-sea Antarctic plankton. Nature. 2001;409(6820):603–607. 10.1038/35054537 11214316

[6] Kan Z, Jaiswal BS, Stinson J, Janakiraman V, Bhatt D, Stern HM, et al Diverse somatic mutation patterns and pathway alterations in human cancers. Nature. 2010;466(7308):869–873. 10.1038/nature09208 20668451

[7] Boyd SD, Marshall EL, Merker JD, Maniar JM, Zhang LN, Sahaf B, et al Measurement and clinical monitoring of human lymphocyte clonality by massively parallel VDJ pyrosequencing. Science Translational Medicine. 2009;1(12):12ra23 10.1126/scitranslmed.3000540 20161664 PMC2819115

[8] Editorial. Your Microbes, Your Health. Science. 2013;342(6165):1440–1441. 10.1126/science.342.6165.1440-b 24357292

[9] Di Bella JM, Bao Y, Gloor GB, Burton JP, Reid G. High throughput sequencing methods and analysis for microbiome research. Journal of Microbiological Methods. 2013;95(3):401–414. 10.1016/j.mimet.2013.08.011 24029734

[10] Mande SS, Mohammed MH, Ghosh TS. Classification of metagenomic sequences: methods and challenges. Briefings in Bioinformatics. 2012;13(6):669–681. 10.1093/bib/bbs054 22962338

[11] Dröge J, McHardy AC. Taxonomic binning of metagenome samples generated by next-generation sequencing technologies. Briefings in Bioinformatics. 2012;13(6):646–655. 10.1093/bib/bbs031 22851513

[12] Li W, Godzik A. Cd-hit: a fast program for clustering and comparing large sets of protein or nucleotide sequences. Bioinformatics. 2006;22(13):1658–1659. 10.1093/bioinformatics/btl158 16731699

[13] Edgar RC. Search and clustering orders of magnitude faster than BLAST. Bioinformatics. 2010;26(19):2460–1. 10.1093/bioinformatics/btq461 20709691

[14] Sun Y, Cai Y, Huse SM, Knight R, Farmeric WG, Wang X, et al A large-scale benchmark study of existing algorithms for taxonomy-independnet microbial community analysis. Briefings in Bioinformatics. 2011;13(1):107–121. 10.1093/bib/bbr009 21525143 PMC3251834

[15] Chen W, Cheng Y, Zhang C, Zhang S, Zhao H. MSClust: A multi-seeds based clustering algorithm for microbiome profiling using 16S rRNA sequences. Journal of Microbiological Methods. 2013;94(3):347–355. 10.1016/j.mimet.2013.07.004 23899776 PMC3895816

[16] Bonder MJ, Abeln S, Zaura E, Brandt BW. Comparing clustering and pre-processing in taxonomy analysis. Bioinformatics. 2012;28(22):2891–2897. 10.1093/bioinformatics/bts552 22962346

[17] Peterson J, Garges S, Giovanni M, Mclnnes P, Wang L, Schloss JA, et al The NIH Human Microbiome Project. Genome Research. 2009;19(12):2317–2323. 10.1101/gr.096651.109 19819907 PMC2792171

[18] Cai Y, Sun Y. ESPRIT-Tree: Hierarchical clustering analysis of millions of 16S rRNA Pyrosequences in quasilinear computational time. Nuclear Acids Research. 2011;39(14):e95 10.1093/nar/gkr349 PMC3152367 21596775

[19] Wang X, Cai Y, Sun Y, Knight R, Mai V. Secondary structure information does not improve OTU assignment for partial 16S rRNA sequences. The ISME Journal. 2012;6(7):1277 10.1038/ismej.2011.187 22170430 PMC3379628

[20] Barriuso J, Valverde JR, Mellado RP. Estimation of bacterial diversity using next generation sequencing of 16S rDNA: a comparison of different workflows. BMC Bioinformatics. 2011;12(1):473 10.1186/1471-2105-12-473 22168258 PMC3258296

[21] Olson CF. Parallel algorithms for hierarchical clustering. Parallel Computing. 1995;21(8):1313–1325. 10.1016/0167-8191(95)00017-I

[22] Dash M, Petrutiu S, Scheuermann P. Efficient parallel hierarchical clustering In: Euro-Par 2004 Parallel Processing. Springer; 2004 p. 363–371.

[23] Feng Z, Zhou B, Shen J. A parallel hierarchical clustering algorithm for PCs cluster system. Neurocomputing. 2007;70(4):809–818. 10.1016/j.neucom.2006.10.034

[24] Rodrigues JFM, von Mering C. HPC-CLUST: distributed hierarchical clustering for large sets of nucleotide sequences. Bioinformatics. 2014;30(2):287–288. 10.1093/bioinformatics/btt657 24215029 PMC3892691

[25] Sun Y, Cai Y, Liu L, Yu F, Farrell ML, McKendree W, et al ESPRIT: estimating species richness using large collections of 16S rRNA pyrosequences. Nuclear Acids Research. 2009;37(10):e76 10.1093/nar/gkp285 PMC2691849 19417062

[26] Nguyen TD, Schmidt B, Zheng Z, Kwoh CF. Efficient and Accurate OTU Clustering with GPU-Based Sequence Alignment and Dynamic Dendrogram Cutting. IEEE/ACM Transactions on Computational Biology and Bioinformatics. 2015;12:1060–1073. 10.1109/TCBB.2015.2407574 26451819

[27] Mao Q, Zheng W, Wang L, Cai Y, Mai V, Sun Y. Parallel Hierarchical Clustering in Linearithmic Time for Large-Scale Sequence Analysis. In: 2015 IEEE International Conference on Data Mining; 2015. p. 310–319.

[28] Edgar RC. MUSCLE: multiple sequence alignment with high accuracy and high throughput. Nucleic Acids Research. 2004;32(5):1792–1797. 10.1093/nar/gkh340 15034147 PMC390337

[29] Katoh K, Misawa K, Kuma K, Miyata T. MAFFT: a novel method for rapid multiple sequence alignment based on fast Fourier transform. Nucleic Acids Research. 2002;30(14):3059–3066. 10.1093/nar/gkf436 12136088 PMC135756

[30] Price MN, Dehal PS, Arkin AP. FastTree 2–approximately maximum-likelihood trees for large alignments. PLoS ONE. 2010;5(3):e9490 10.1371/journal.pone.0009490 20224823 PMC2835736

[31] Howe K, Bateman A, Durbin R. QuickTree: building huge Neighbour-Joining trees of protein sequences. Bioinformatics. 2002;18(11):1546–1547. 10.1093/bioinformatics/18.11.1546 12424131

[32] Quinn MJ. Parallel Programming in C with MPI and OpenMP. Boston: McGraw-Hill; 2004.

[33] Skiena S. The Algorithm Design Manual. Springer; 2008.

[34] Edgar RC, Haas BJ, Clemente JC, Quince C, Knight R. UCHIME improves sensitivity and speed of chimera detection. Bioinformatics. 2011;27(16):2194–2200. 10.1093/bioinformatics/btr381 21700674 PMC3150044

[35] Edgar RC. UPARSE: Highly accurate OTU sequences from microbial amplicon reads. Nature Methods. 2013;10:996–998. 10.1038/nmeth.2604 23955772

[36] Turnbaugh PJ, Hamady M, Yatsunenko T, Cantarel BL, Duncan A, Ley RE, et al A core gut microbiome in obese and lean twins. Nature. 2008;457(7228):480–484. 10.1038/nature07540 19043404 PMC2677729

[37] Ye J, McGinnis S, Madden TL. BLAST: improvements for better sequence analysis. Nucleic acids research. 2006;34(suppl 2):W6–W9. 10.1093/nar/gkl164 16845079 PMC1538791

[38] Cole JR, Chai B, Farris RJ, Wang Q, Kulam S, McGarrell DM, et al The Ribosomal Database Project (RDP-II): sequences and tools for high-throughput rRNA analysis. Nucleic acids research. 2005;33(suppl 1):D294–D296. 10.1093/nar/gki038 15608200 PMC539992

[39] Giongo A, Davis-Richardson AG, Crabb DB, Triplett EW. TaxCollector: modifying current 16S rRNA databases for the rapid classification at six taxonomic levels. Diversity. 2010;2(7):1015–1025. 10.3390/d2071015

[40] Claesson MJ, Cusack S, O’Sullivan O, Greene-Diniz R, de Weerd H, Flannery E, et al Composition, variability, and temporal stability of the intestinal microbiota of the elderly. Proceedings of the National Academy of Sciences. 2011;108(1):4586–4591. 10.1073/pnas.1000097107 PMC3063589 20571116

[41] Human Microbiome Project Consortium. Structure, function and diversity of the healthy human microbiome. Nature. 2012;486(7402):207–214. 10.1038/nature11234 22699609 PMC3564958

[42] Ding T, Schloss PD. Dynamics and associations of microbial community types across the human body. Nature. 2014;509(7500):357–360. 10.1038/nature13178 24739969 PMC4139711

[43] Koeppel AF, Wu M. Surprisingly extensive mixed phylogenetic and ecological signals among bacterial Operational Taxonomic Units. Nucleic acids research. 2013; p. gkt241. 10.1093/nar/gkt241 PMC3664822 23571758

[44] Westcott SL, Schloss PD. De novo clustering methods outperform reference-based methods for assigning 16S rRNA gene sequences to operational taxonomic units. PeerJ. 2015;3:e1487 10.7717/peerj.1487 26664811 PMC4675110

[45] May A, Abeln S, Crielaard W, Heringa J, Brandt BW. Unraveling the outcome of 16S rDNA-based taxonomy analysis through mock data and simulations. Bioinformatics. 2014;30(11):1530–1538. 10.1093/bioinformatics/btu085 24519382

[46] Flynn JM, Brown EA, Chain FJ, MacIsaac HJ, Cristescu ME. Toward accurate molecular identification of species in complex environmental samples: testing the performance of sequence filtering and clustering methods. Ecology and evolution. 2015;5(11):2252–2266. 10.1002/ece3.1497 26078860 PMC4461425

[47] White JR, Navlakha S, Nagarajan N, Ghodsi MR, Kingsford C, Pop M. Alignment and clustering of phylogenetic markers-implications for microbial diversity studies. BMC bioinformatics. 2010;11(1):1 10.1186/1471-2105-11-152 20334679 PMC2859756

[48] Wang X, Yao J, Sun Y, Mai V. M-pick, a modularity-based method for OTU picking of 16S rRNA sequences. BMC bioinformatics. 2013;14(1):1 10.1186/1471-2105-14-43 23387433 PMC3599145

[49] Lozupone C, Knight R. UniFrac: a new phylogenetic method for comparing microbial communities. Applied and environmental microbiology. 2005;71(12):8228–8235. 10.1128/AEM.71.12.8228-8235.2005 16332807 PMC1317376

[50] Corpet F. Multiple sequence alignment with hierarchical clustering. Nucleic acids research. 1988;16(22):10881–10890. 10.1093/nar/16.22.10881 2849754 PMC338945

[51] Krause A, Stoye J, Vingron M. Large scale hierarchical clustering of protein sequences. BMC bioinformatics. 2005;6(1):1 10.1186/1471-2105-6-15 15663796 PMC547898

