# Supplementary material for: ESPRIT-Forest: Parallel clustering of massive amplicon sequence data in subquadratic time
Source: PLoS Comput Biol. 2017 Apr 24;13(4):e1005518. doi: 10.1371/journal.pcbi.1005518 (PMC5421816; doi:10.1371/journal.pcbi.1005518)
Supplement: S1 Methods — (PDF) [file pcbi.1005518.s001.pdf]

S1 Supporting Methods: Theoretical Proof for the Equivalence of Parallel Hierarchical Clustering to Sequential Ones

This section describes the theoretical basis of ESPRIT-Forest. Firstly, we mathematically define the concept of hierarchical clustering and introduce a few definitions used in this section. Let $\mathcal{D} = \{x_1, x_2, \ldots, x_N\}$ be a dataset containing N samples (sequences) and $d(x_i, x_j)$ be a distance measuring the similarity between two samples x_i and x_j.

Definition 1 (Cluster). *A set containing a single sample forms a cluster. If $\mathcal{S}_1$ and $\mathcal{S}_2$ are two clusters, then $\mathcal{S} = \mathcal{S}_1 \cup \mathcal{S}_2$ is also a cluster.*

Definition 2 (Distance between Clusters). *Let $\mathcal{S}_1$ and $\mathcal{S}_2$ be two clusters. A binary function $D(\mathcal{S}_1, \mathcal{S}_2)$ is a distance function of two clusters if the following properties are satisfied:*

1. *If both $\mathcal{S}_1$ and $\mathcal{S}_2$ contain only one sample, i.e., $\mathcal{S}_1 = \{x_1\}$ and $\mathcal{S}_2 = \{x_2\}$, then $D(\mathcal{S}_1, \mathcal{S}_2) = d(x_1, x_2)$;*
2. *$D(\mathcal{S}_1, \mathcal{S}_2) = D(\mathcal{S}_2, \mathcal{S}_1)$;*
3. *If $\mathcal{S}_2 = \mathcal{S}_a \cup \mathcal{S}_b$, then there exists a binary function f so that $D(\mathcal{S}_1, \mathcal{S}_2) = f(D(\mathcal{S}_1, \mathcal{S}_a), D(\mathcal{S}_1, \mathcal{S}_b))$ and $f(x, y) \geq 0$ for any x and y.*

The above definition states that a distance between clusters should be symmetric and consistent with a sample distance. Also, a distance between clusters can be derived from the distances between their sub-clusters.

We further assume that the distance function meet the following non-decreasing property, which is the case in most current sequence clustering algorithms, including average-link, single-link and complete-link clustering, as well as centroid-link clustering using profile-profile alignments.

Definition 3 (Non-Decreasing Property). *Let $D(\cdot)$ be a distance function between clusters. $D(\cdot)$ is said to have a non-decreasing property if $D(\mathcal{S}_1, \mathcal{S}_2) \geq \min(D(\mathcal{S}_1, \mathcal{S}_a), D(\mathcal{S}_1, \mathcal{S}_b))$, for any clusters $\mathcal{S}_1, \mathcal{S}_a, \mathcal{S}_b$ and $\mathcal{S}_2 = \mathcal{S}_a \cup \mathcal{S}_b$.*

For convenience of statement, we formulate the usual concept of nearest neighbor, which is the basis of hierarchical clustering, as follows:

Definition 4 (Nearest Neighbor). *Let Ω be the indices of clusters in a clustering step, and $\Theta = \{\mathcal{S}_i\}_{i \in \Omega}$ be a set of clusters. For any $i \in \Omega$, the nearest neighbor of $\mathcal{S}_i$ is defined as $\mathrm{NN}_\Omega(\mathcal{S}_i) ::= \mathrm{argmin}_{j \in \Omega, j \neq i} D(\mathcal{S}_i, \mathcal{S}_j)$.*

We further define two operators $\sqsubseteq$ and $\preceq$, which will be frequently met in clustering, as follows:

Definition 5 (Derived Clusters)**.** *Let $\mathcal{S}$ be a cluster and Ω be the indices of a set of clusters. We denote $\mathcal{S} \sqsubseteq \Omega$ and $\Delta \preceq \mathcal{S}$ if there exists a subset $\Delta = \{a_1, a_2, \cdots, a_m\} \subseteq \Omega$ so that $\mathcal{S} = \mathcal{S}_{a_1} \cup \mathcal{S}_{a_2} \cup \cdots \cup \mathcal{S}_{a_m}$.*

Let $\mathcal{D} = \{x_1, x_2, \ldots, x_N\}$ be a dataset containing N samples (sequences), $\{\mathcal{S}_i\}$ be the obtained clusters and $D(\mathcal{S}_1, \mathcal{S}_2)$ be a binary function defining the distance between any two clusters, a hierarchical clustering algorithm is formally described in Algorithm 1.

Algorithm 1: Hierarchical Clustering

Input: $\mathcal{D} = \{x_n\}_{n=1}^{N}$, stop criterion d_{up}, distance function $D(\cdot)$;
Initialization: $\mathcal{S}_n = \{x_n\}, 1 \leq n \leq N, \Omega_0 = \{1, \ldots, N\}$, $k = 0$;
repeat
  $\{a, b\} = \mathrm{argmin}_{i,j \in \Omega_k, i \neq j} D(\mathcal{S}_i, \mathcal{S}_j)$;
  $k = k + 1$;
  $\mathcal{S}_{N+k} = \mathcal{S}_a \cup \mathcal{S}_b$;
  $\Omega_k = \Omega_{k-1} \cup \{N + k\} \setminus \{a, b\}$;
until $|\Omega_k| = 1$ or $\min_{i,j \in \Omega_k, i \neq j} D(\mathcal{S}_i, \mathcal{S}_j) \geq d_{up}$;
Output: A set of generated clusters $\{\mathcal{S}_1, \cdots, \mathcal{S}_{N+k}\}$ and a set of existing clusters Ω_k.

The key challenge of parallel merging is to design a clustering scheme that produces results identical to that generated by standard hierarchical clustering, which is important in order to preserve clustering quality. We demonstrate below that by using a proper merging criterion, multi-point clustering can achieve a clustering result equivalent to that of standard single-point hierarchical clustering. Specifically, in each step, we can merge all pairs $\{a, b\}$ that satisfy $\mathrm{NN}(a) = b$ and $\mathrm{NN}(b) = a$ (Here NN denotes the nearest neighbour), without violating the rule used conventional hierarchical clustering. The modified algorithm is described in Algorithm 2.

Algorithm 2: Multi-point Hierarchical Clustering

Input: $\mathcal{D} = \{x_n\}_{n=1}^{N}$, stop criterion d_{up}, distance function $D(\cdot)$;
Initialization: $\mathcal{S}_n = \{x_n\}, 1 \leq n \leq N, \Omega_0 = \{1, \ldots, N\}$, $k = 0$;
repeat
  Set $\Phi = \{\{a_i, b_i\} \mid \mathrm{NN}_{\Omega_k}(\mathcal{S}_{a_i}) = b_i, \mathrm{NN}_{\Omega_k}(\mathcal{S}_{b_i}) = a_i, D(\mathcal{S}_{a_i}, \mathcal{S}_{b_i}) < d_{up}\}$;
  for $i \leftarrow 1$ **to** $|\Phi|$ **do**
    $k = k + 1$;
    $\mathcal{S}_{N+k} = \mathcal{S}_{a_i} \cup \mathcal{S}_{b_i}$;
    $\Omega_k = \Omega_{k-1} \cup \{N + k\} \setminus \{a_i, b_i\}$;
until $|\Omega_k| = 1$ or $\min_{i,j \in \Omega_k, i \neq j} D(\mathcal{S}_i, \mathcal{S}_j) \geq d_{up}$;
Output: A set of generated clusters $\{\mathcal{S}_1, \cdots, \mathcal{S}_{N+k}\}$ and a set of existing clusters Ω_k.

The following theorem formally states that the parallel hierarchical clustering method adopted by ESPRIT-Forest generates consistent result with standard hierarchical clustering:

Theorem 1. *Let $D(\cdot)$ be a distance function satisfying the non-decreasing property. Let $\{\mathcal{S}_k\}_{k=1}^K$ be a series of clusters generated by Algorithm 2, and $\{d_k\}_{k=1}^K$ be the corresponding NN pair distances at each step. By sorting $\{d_k\}$ in an ascending order $\{d_{s_j}\}_{j=1}^K$ so that $d_{s_i} \le d_{s_j}$ for all $i < j$, then $\{\mathcal{S}_{s_j}\}_{j=1}^K$ are also a series of clusters generated by Algorithm 1.*

We first prove Theorem 1 with an additional restriction that in any step of hierarchical clustering the nearest neighbor is unique for all existing clusters and thus the solution of hierarchical clustering is unique. Then, we will go on to prove that such a restriction can be relaxed and Theorem 1 holds in general cases. To achieve the first goal, two lemmas are established. Lemma 1 states that a multi-point merging operation executed in any clustering step of Algorithm 1 achieves the same result as the original algorithm. Lemma 2 shows that the multi-point merging operations can be executed iteratively while maintaining a clustering result identical to that obtained by the original algorithm.

Lemma 1. *Let $\{i, j\}$ be a pair of clusters that are generated in step k of Algorithm 1 (including the initial step). If the following conditions hold:*

1. *The distance function $D(\cdot)$ satisfies the non-decreasing property;*
2. *For any step $\bar{k}$ in Algorithm 1 and any $\bar{i} \in \Omega_{\bar{k}}$, $\mathrm{NN}_{\Omega_{\bar{k}}}(\mathcal{S}_{\bar{i}})$ is unique;*
3. $\mathrm{NN}_{\Omega_k}(\mathcal{S}_i) = j$ *and* $\mathrm{NN}_{\Omega_k}(\mathcal{S}_j) = i$ *and* $D(\mathcal{S}_i, \mathcal{S}_j) \le d_{up}$,

then there exists a step k' in Algorithm 1 so that $\mathcal{S}_{k'} = \mathcal{S}_i \cup \mathcal{S}_j$.

Proof. If such a k' does not exist, in order not to violate the stopping criteria of hierarchical clustering, at least one of the following two cases must be true: (i) there exists a step k'' so that $\mathcal{S}_{k''} = \mathcal{S}_i \cup \mathcal{S}_{j_0}$ and $j_0 \ne j$, with $j \in \Omega_{k''}$; (ii) there exists a step k'' so that $\mathcal{S}_{k''} = \mathcal{S}_{i_0} \cup \mathcal{S}_j$ and $i_0 \ne i$, with $i \in \Omega_{k''}$.

Without loss of generality, we consider the first case (the proof of the second case is essentially identical). We have $\{i, j_0\} = \mathrm{argmin}_{x,y \in \Omega_{k''}, x \ne y}\, D(\mathcal{S}_x, \mathcal{S}_y)$. It follows that $\mathrm{NN}_{\Omega_{k''}}(\mathcal{S}_i) = j_0$. On the other hand, we have $\mathrm{NN}_{\Omega_k}(\mathcal{S}_i) = j$. Thus, $\min_{x \in \Omega_k \setminus i} D(\mathcal{S}_i, \mathcal{S}_x) = D(\mathcal{S}_i, \mathcal{S}_j)$.

Let $\Omega_M = \Omega_k \cap \Omega_{k''}$, $\Delta_k = \Omega_k \setminus \Omega_M$ and $\Delta_{k''} = \Omega_{k''} \setminus \Omega_M$. From the property of Algorithm 1, we know that $i \in \Omega_M$ and $j \in \Omega_M$. Moreover, for any element $x \in \Delta_{k''}$, we have $\mathcal{S}_x \sqsubseteq \Delta_k$. Therefore, by using the non-decreasing property of the cluster distances, we have

$$
\begin{aligned}
&\min_{x \in \Omega_{k''}} D(\mathcal{S}_i, \mathcal{S}_x) \\
=&\min\left(\min_{x \in \Omega_M \setminus i} D(\mathcal{S}_i, \mathcal{S}_x),\ \min_{x \in \Delta_{k''}} D(\mathcal{S}_i, \mathcal{S}_x)\right) \\
\ge&\min\left(\min_{x \in \Omega_M \setminus i} D(\mathcal{S}_i, \mathcal{S}_x),\ \min_{x \in \Delta_k} D(\mathcal{S}_i, \mathcal{S}_x)\right) \\
=&\min_{x \in \Omega_k \setminus i} D(\mathcal{S}_i, \mathcal{S}_x) = D(\mathcal{S}_i, \mathcal{S}_j)\ .
\end{aligned}
$$

It means that $\mathrm{NN}_{\Omega_{k''}}(\mathcal{S}_i) = j$, which violates the second condition of Lemma 1. Hence, k'' does not exist and k' must exist. ■ □

Lemma 1 states that by taking a multi-point merging step, we merely changes the order of the generated clusters but will not create a cluster that is incompatible with the original clustering result. In Lemma 2, we go on to prove that by allowing multiple clusters to be formed prematurely, the clustering result is the same as the original one.

Lemma 2. *Let $\Omega = \{k_i\}_{i=1}^m$ be a set of cluster indices where each cluster $\mathcal{S}_{k_i}$ is formed in step k_i of Algorithm 1, $\mathcal{S}_{k_i} \cap \mathcal{S}_{k_j} = \phi$ for any $i \neq j$, and $\bigcup_{i=1}^m \mathcal{S}_{k_i} = \{x_1, .., x_N\}$ (i.e., the clusters listed in Ω cover the entire data set). Without loss of generality, let i and j be the indices of two arbitrary clusters in Ω, if the following conditions hold:*

1. *The distance function $D(\cdot)$ satisfies the non-decreasing property;*
2. *For any step $\bar{k}$ in Algorithm 1 and any $\bar{i} \in \Omega_{\bar{k}}$, $\mathrm{NN}_{\Omega_{\bar{k}}}(\mathcal{S}_{\bar{i}})$ is unique;*
3. *$\mathrm{NN}_\Omega(\mathcal{S}_i) = j$ and $\mathrm{NN}_\Omega(\mathcal{S}_j) = i$ and $D(\mathcal{S}_i, \mathcal{S}_j) < d_{up}$;*

then there exists a step k in Algorithm 1 so that $\mathcal{S}_k = \mathcal{S}_i \cup \mathcal{S}_j$.

Proof. If such a k does not exist, at least one of the following two cases must be true in Algorithm 1 : (i) there exists a step k'_1 so that $\mathcal{S}_{k'_1} = \mathcal{S}_i \cup \mathcal{S}_{j_0}$ and $j_0 \neq j$; (ii) there exists a step k'_2 so that $\mathcal{S}_{k'_2} = \mathcal{S}_{i_0} \cup \mathcal{S}_j$ and $i_0 \neq i$. Without loss of generality, we consider the case where (i) occurs before (ii)(the proof of the reverse case is essentially identical).

j may or may not exist in $\Omega_{k'_1}$. Nevertheless, there must exist $\mathcal{S}_j \sqsubseteq \Omega_{k'_1}$; otherwise it means that $\mathcal{S}_j$ has been merged before step k'_1, which violates the assumption that (i) occurs first. Let $\Delta_{k'_1}$ be the subset of $\Omega_{k'_1}$ so that $\Delta_{k'_1} \preceq \mathcal{S}_j$. We have $\min_{x\in\Delta_{k'_1}} \mathrm{D}(\mathcal{S}_i, \mathcal{S}_x) \leq D(\mathcal{S}_i, \mathcal{S}_j)$ according to the non-decreasing property of D. Hence, it must hold that $D(\mathcal{S}_i, \mathcal{S}_{j_0}) < D(\mathcal{S}_i, \mathcal{S}_j)$; otherwise it violates the assumption that $\mathcal{S}_{j_0}$ is the unique NN of $\mathcal{S}_i$.

We now check the relationship between $\mathcal{S}_{j_0}$ and Ω. For any element $x \in \mathcal{S}_{j_0}$, we can find a corresponding cluster index t_x in Ω so that $x \in \mathcal{S}_{t_x}$ because the clusters listed in Ω cover the entire data set. Following the rule of hierarchical clustering, we have either $\mathcal{S}_{t_x} \subseteq \mathcal{S}_{j_0}$ or $\mathcal{S}_{j_0} \subset \mathcal{S}_{t_x}$. Since $\mathcal{S}_{t_x}$ is a cluster generated in Algoirthm 1, the latter case cannot happen because it means that $\mathcal{S}_{j_0}$ is merged into a cluster different from $\mathcal{S}_i$, which violates the uniqueness of the solution. With all $\mathcal{S}_{t_x} \subseteq \mathcal{S}_{j_0}$ (and hence $\mathcal{S}_{j_0} \sqsubseteq \Omega$), we have $\min_{x\in\Omega} D(\mathcal{S}_i, \mathcal{S}_x) \leq \min_{x\in\mathcal{S}_{j_0}} D(\mathcal{S}_i, \mathcal{S}_{t_x}) \leq D(\mathcal{S}_i, \mathcal{S}_{j_0}) < D(\mathcal{S}_i, \mathcal{S}_j)$, which violates the assumption that $\mathrm{NN}_\Omega(\mathcal{S}_i) = j$. Hence, j_0 cannot exist and k must exist. ■ □

By Lemmas 1 and 2, we confirm that under the assumption that the nearest neighbor is unique, every cluster generated in Algorithm 2 should also be generated in Algorithm 1. In Lemma 3, we go on to prove that every cluster generated in Algorithm 1 should also be generated in Algorithm 2, and thus Theorem 1 holds with an additional restriction that the NN solutions are always unique.

Lemma 3. *Given conditions 1 and 2 in Lemma 1, for any cluster $\mathcal{S}$ in the final result of Algorithm 1, $\mathcal{S}$ also exists in the final result of Algorithm 2.*

Proof. Suppose there exists a cluster $\mathcal{S}_k$ in the final result of Algorithm 1 that is not in the result of Algorithm 2. The step of generating $\mathcal{S}_k$ can be described as $\mathcal{S}_k = \mathcal{S}_a \cup \mathcal{S}_b$. Let Ω be the set of clusters that exists in the final result of Algorithm 2. It must hold that $\mathcal{S}_a \sqsubseteq \Omega$ and $\mathcal{S}_b \sqsubseteq \Omega$; otherwise there would be a cluster generated in Algorithm 2 but not in Algorithm 1, which contradicts lemma 2. Let Φ_a and Φ_b be the corresponding subsets of Ω so that $\Phi_a \preceq \mathcal{S}_a$ and $\Phi_b \preceq \mathcal{S}_b$. Using the non-decreasing property of $D(\cdot)$, we have $\min_{i\in\Phi_a, j\in\Phi_b} D(\mathcal{S}_i, \mathcal{S}_j) \leq D(\mathcal{S}_a, \mathcal{S}_b) \leq d_{up}$. This in turn leads to a contradiction that the terminal condition of Algorithm 2 has not been met yet. Hence, $\mathcal{S}_k$ does not exist.■ □

We next prove that the uniqueness of nearest neighbors is not necessary for the two algorithms to be equivalent, which concludes the proof of Theorem 1.

Proof of Theorem 1. We first prove that in case of non-unique NN, we can carry out multi-point merging and maintain a clustering result consistent with the original one by dividing the clustering procedure into various stages. Without loss of generality, we can assume that in Algorithm 1, the uniqueness of NN holds for all the first $k_1 - 1$ merging steps, but in step k_1 a tie occurs for cluster i in $\mathrm{NN}_{\Omega_{k_1}}(\mathcal{S}_i)$, with $D(\mathcal{S}_i, \mathrm{NN}_{\Omega_{k_1}}(\mathcal{S}_i)) = d_1$. We call k_1 a tie-point and d_1 a tie-point distance. Suppose that we execute Algorithm 2 on the same data using $d_{up} = d_1$ as the stopping criterion. By applying Lemma 2, the clusters generated by Algorithm 2 is thus identical to the first $k - 1$ steps of Algorithm 1. Then, by merging cluster i with either one of its NNs, we get a clustering result that is identical to one of the possible solutions in the first k_1 steps of Algorithm 1. Suppose that the next tie-point for Algorithm 1 is k_2 with distance d_2. We then go on to carry out Algorithm 2 with $d_{up} = d_2$ as the stopping criterion. By applying Lemma 2 again, we can still get a clustering solution that is one solution of Algorithm 1 in the first k_2 steps. Hence, by splitting the clustering procedure into multiple stages according to each tie-point, we can apply Algorithm 2 within each stage and get the same result as Algorithm 1.

Finally, we show that the above multi-stage splitting can be relaxed. Let k_u be tie-point with d_u and d_d being the largest and second largest tie-point distances (if such a tie-point does not exist, we can set $d_d = 0$), respectively, and k_d the first step beyond that tie-point. We consider the following set of cluster indices $\Omega = \{k_i\}_{i=1}^m$ where each cluster $\mathcal{S}_{k_i}$ is formed in step k_i of Algorithm 1, with either $k_i \in \Omega_{k_d}$ or $k_i > k_d$. Moreover, $\mathcal{S}_{k_i} \cap \mathcal{S}_{k_j} = \phi$ for any $i \neq j$, and $\bigcup_{i=1}^m \mathcal{S}_{k_i} = \{x_1, .., x_N\}$. Hence, Ω represents a possible result of carrying out Algorithm 2 with Ω_{k_d} as the starting point.

Let i and j be the indices of two arbitrary clusters in Ω, where $\mathrm{NN}_\Omega(\mathcal{S}_i) = j$, $\mathrm{NN}_\Omega(\mathcal{S}_j) = i$ and $D(\mathcal{S}_i, \mathcal{S}_j) < d_{up}$. We now check if there always exists a step k in Algorithm 1 so that $\mathcal{S}_k = \mathcal{S}_i \cup \mathcal{S}_j$. Suppose $i \in \Omega_{k_u}$ and $j \in \Omega_{k_u}$, or reversely $i \notin \Omega_{k_u}$ and $j \notin \Omega_{k_u}$. The situation can then be transferred to applying Algorithm 2 within one stage defined in the first paragraph of this proof, and hence the desired step k must exist. We then consider the case where one of i, j is not in Ω_{k_u}. Without loss of generality, we can set $i \notin \Omega_{k_u}$ but $j \in \Omega_{k_u}$. By definition, there should exist a step $k_d < k' < k_u$

with $\mathcal{S}_{k'} = \mathcal{S}_i \cup \mathcal{S}_{j_0}$ and $j_0 \neq j$. Similar to the proof of Lemma 2, we have $\mathcal{S}_j \sqsubseteq \Omega_{k'}$. Furthermore, by definition, $j_0 \notin \Omega_{k_u}$ and there is no tie-point between k_d and k_u. Thus, the unique property of NN solution should not be violated for j_0. Hence, we also have $\min_{x\in\Omega} D(\mathcal{S}_i, \mathcal{S}_x) \leq D(\mathcal{S}_i, \mathcal{S}_{j_0}) < D(\mathcal{S}_i, \mathcal{S}_j)$ following the same steps in Lemma 2, which violates $\text{NN}_\Omega(\mathcal{S}_i) = j$. Thus, j_0 cannot exist and k must exist. It follows that Algorithm 2, starting from Ω_{k_d}, can be carried out regardless of the tie-point d_u and produces the same result as Algorithm 1.

By applying the same reasoning on all tie-points in a top-down order, we prove that Algorithm 2 always produces a result consistent with Algorithm 1 even in the presence of tie-points. This concludes the proof of Theorem 1. ■ □
